# *RAN1* is involved in plant cold resistance and development in rice (*Oryza sativa*)

**DOI:** 10.1093/jxb/eru178

**Published:** 2014-04-30

**Authors:** Peipei Xu, Weiming Cai

**Affiliations:** Institute of Plant Physiology and Ecology, Shanghai Institutes for Biological Sciences, Graduate School of Chinese Academy of Sciences, Chinese Academy of Sciences, 300 Fenglin Road, Shanghai 200032, China

**Keywords:** Cold tolerance, cell division, *OsRAN1*, reduced apical dominance, intact nuclear envelope.

## Abstract

Of the diverse abiotic stresses, low temperature is one of the major limiting factors that lead to a series of morphological, physiological, biochemical, and molecular changes in plants. Ran, an evolutionarily conserved small G-protein family, has been shown to be essential for the nuclear translocation of proteins. It also mediates the regulation of cell cycle progression in mammalian cells. However, little is known about Ran function in rice (*Oryza sativa*). We report here that Ran gene *OsRAN1* is essential for the molecular improvement of rice for cold tolerance. Ran also affects plant morphogenesis in transgenic *Arabidopsis thaliana*. *OsRAN1* is ubiquitously expressed in rice tissues with the highest expression in the spike. The levels of mRNA encoding *OsRAN1* were greatly increased by cold and indoleacetic acid treatment rather than by addition of salt and polyethylene glycol. Further, *OsRAN1* overexpression in *Arabidopsis* increased tiller number, and altered root development. *OsRAN1* overexpression in rice improves cold tolerance. The levels of cellular free Pro and sugar levels were highly increased in transgenic plants under cold stress. Under cold stress, *OsRAN1* maintained cell division and cell cycle progression, and also promoted the formation of an intact nuclear envelope. The results suggest that OsRAN1 protein plays an important role in the regulation of cellular mitosis and the auxin signalling pathway.

## Summary

*OsRAN1* can improve cold tolerance in rice. It maintains cell division under cold conditions. Overexpression of *OsRAN1* increases tiller number and alters root development.

## Introduction

Ran is a small GTPase that is essential for nuclear transport, nuclear assembly, mRNA processing, and cell cycle control, and is also the only known member of the family of small GTP-binding proteins primarily localized inside the nucleus (Ciciarello *et al.*, 2007; Di Fiore *et al.*, 2004). Ran can switch between a GDP- and GTP-bound state, and the transition from RanGDP to RanGTP can occur by nucleotide exchange. Although the intrinsic rate of nucleotide exchange and hydrolysis of Ran is slow, it is stimulated by the regulator of chromosome condensation 1 (RCC1) and the GTPase-activating protein (RanGAP1) (Gorlich and Kutay, 1999; Sazer and Dasso, 2000). As RanGAP and RanBP1 (Ran binding protein 1) are excluded from the nucleus, they cooperate in the cytoplasm to deplete RanGTP (Bischoff and Ponstingl, 1991; Bischoff and Ponstingl, 1995; Ramdas *et al.*, 1991). In plants, the identification of RanBP1 and RanGAP enabled the study of Ran function (Ach and Gruissem, 1994; Rose and Meier, 2001). Owing to the high similarity in amino acid sequence and subcellular localization, plant Ran proteins are probably highly conserved with their mammalian and yeast counterparts in nucleo-cytoplasmic trafficking and mitotic processes (Wang *et al.*, 2006).

Ran is encoded by a family of four genes in *Arabidopsis* and three genes in rice (Haizel *et al.*, 1997; Vernoud *et al.*, 2003). The temperature-sensitive mutants of *Pim1* (premature initiation of mitosis 1) in fission yeast enter mitosis without completing chromosomal DNA replication, and overexpression of yeast Ran GTPase homologue *Spi1* suppresses the *pim1-46* mutant phenotype (Matsumoto and Beach, 1991). Wheat *RAN1* is involved in regulation of cell division and alters primordial meristem, mitotic progress, and sensitivity to auxin in rice and *Arabidopsis* (Wang *et al.*, 2006). Ran GTPase may be involved in the plant response to hormone or environment signalling. Decreased ATP levels induced by oxidative stress lead to decrease in Ran–GTP levels and disordered Ran distribution (Yasuda *et al.*, 2006). The expression of *OsRAN1* is induced by jasmonic acid in rice (Miche *et al.*, 2006); overexpression of *OsRAN2* affects the sensitivity to salt stress in rice (Zang *et al.*, 2010). *OsRAN2* also plays an important role in cold tolerance (Chen *et al.*, 2011). Therefore, Ran protein not only plays an important role in plant development but also mediates plant response to the environment.

Rice is a cold-sensitive plant that has its origin in tropical or sub-tropical areas. Unpredictable cold snaps at the booting stage delay heading and result in pollen sterility owing to the failure of microspore development under low-temperature conditions, which was thought to be one of the key factors responsible for reduced grain yield (Imin *et al.*, 2004). Screening for genes involved in cold tolerance is an important initial step for crop improvement strategy using genetic engineering (Andaya and Tai, 2006; Dai *et al.*, 2004). Maintenance of cell division is essential for plant survival and growth during cold stress. However, the cell cycle-associated cold response requires further research. In this study, we explored the role of *OsRAN1* in the cell cycle and cold tolerance regulation in rice. We also observed the nuclear envelope integrity during cold stress. Our results suggest that plant Ran GTPase may have an important and conserved role in cold stress signalling in plants.

## Materials and methods

### Plant materials

Rice plants (Oryza sativa L. ssp. japonica) were germinated and grown in Hoagland nutrient solution and soil substrate at a photo flux density of 350–400 μmol m^−2^ s^−1^, 60–80% relative humidity, 12/12h day/night cycle at 28 °C in a phytotron. *Arabidopsis* (ecotype Columbia) and tobacco (*Nicotiana tabacum* L. cv. Gexin No.1) were grown under long-day conditions (16/8h light/dark cycle) with a fluence rate of 120 μmol m^−2^ s^−1^ of white light (produced by cool-white fluorescent lamps) at 22 °C. The pot volume was 12×12×10cm. The soil substrate used in the experiments was obtained from the suburban area of Shanghai, China.

### Plasmid constructions and generation of transgenic plants

The full-length cDNA of *OsRAN1* was amplified from rice plant Zhonghua 11 (*Oryza sativa* L. ssp. *japonica*) according to GenBank accession No. AB015971. The modified green fluorescent protein (GFP) gene was amplified using the pCMBIA1302 vector. To construct the plasmid for gene overexpression. *OsRAN1* or *OsRAN1:GFP* were cloned into a pHB vector (Mao *et al.*, 2005) to generate double *35S:OsRAN1* and *35S:OsRAN1:GFP* transgenics. Expression in rice was controlled by the CaMV 35S promoter. The transgenes were confirmed by DNA sequencing. All the reagents and enzymes used here for PCR amplification or restriction digestion were purchased from Takara, Japan. *Arabidopsis* plants (ecotype Columbia) were transformed with double *35S:OsRAN1* and *35S:OsRAN1:GFP* transgenes using the floral-dipping method (Clough and Bent, 1998). Japonica rice cv. Zhonghua11 plants were transformed with the double *35S:OsRAN1:GFP* using an *Agrobacterium*-mediated transformation as described (Hiei *et al.*, 1994). Hygromycin (Roche)-resistance was used to screen positive transgenic plants. Genomic PCR was used to confirm the transgenic plants with specific primers for the hygromycin phosphotransferase (HPT) gene. Semi-quantitative RT-PCR and qPCR were conducted to detect gene expression level in transgenic *Arabidopsis* and rice plants. Primers used for plasmid constructions are shown in Supplementary Table S1 available at *JXB* online.

### Subcellular localization study

Tobacco epidermal cells were injected with the construct of double *35S:OsRAN1:GFP* construct and analysed by confocal microscopy (Zeiss LSM510; Jena, Germany). In addition, the GFP signal was also observed in roots of 1-week-old transgenic *Arabidopsis* seedlings.

### RT-PCR and real-time PCR

The synthesis of cDNA using ReverTra Ace qPCR Master Kit (FSQ-201) was performed as described previously (Zang *et al.*, 2010). An aliquot of 2 μl of tenfold-diluted cDNA was used as an RT-PCR template in a 20 μl reaction system. All PCR products were loaded onto a 1% agarose gel to visualize the amplified cDNAs. RT-PCR was repeated three times. *OsUbiquitin* was used as a control for 25 cycles. Fluorescence intensity of DNA bands was quantified using Bio-Rad’s ChemiDoc MP System. The relative expression level of O*sRAN1* before treatment was set at 1 in different stress condition. For real-time PCR, the cDNA samples were diluted to 2ng μl^−1^. Triplicate quantitative assays were performed with 1 μl of cDNA dilution with the SYBR GreenMaster mix and an ABI fast sequence detection system according to the manufacturer’s protocol (Applied Biosystems, Foster City, CA, USA). The relative quantification method (Delta-Delta CT) was used to evaluate quantitative variation. The amplification of *actin2* was used as an internal control to normalize all data. The primers for gene expression are listed in Supplementary Table S2 available at *JXB* online.

### Stress treatment of rice seedlings

Homologous T2 transgenic rice (*OsRAN1* overexpression) seeds were used for the stress tolerance assay. All seeds were germinated in a mixture of nutrimental soil and vermiculite (2:1). Transgenic and wild-type seedlings were grown under 12h light/12h dark (28 °C/25 °C). To search the expression pattern of OsRAN1, one-week-old transgenic rice seeds were germinated in water (as control) or in water containing 150mM NaCl, 10% PEG 6000 (polyethylene glycol, average Mn 6000), or 1 μM indoleacetic acid (IAA). For cold treatment, two-week-old seedlings at the trefoil stage were treated at 4 °C for 84h under 12h light/12h dark. After treatment, the seedlings were moved to a greenhouse for recovery in 2 weeks. Photographs were taken at the indicated times.

### Imaging of root cell size

To examine cell arrangement and size, root tips were stained with 100 μg μl^–1^ propidium iodide solution and observed under a confocal laser scanning microscope (Zeiss) with an argon laser.

### Measurement of Pro and soluble sugar contents

Transgenic plants at the four-leaf stage were used for biochemical analysis. Free Pro content in leaves was determined by established techniques (Troll and Lindsley, 1955). About 50mg of leaves were homogenized in 10ml of sulfosalicylic acid (3%) and the homogeneous mixture was centrifuged at 13 000rpm for 15min at 4 °C. The extract (2ml) was transformed to a microcentrifuge and mixed with 2ml of acid ninhydrin (0.1g ninhydrin dissolved in 24ml of glacial acetic acid and 16ml of 6-mortho-phosphoric acid) and 2ml of acetic acid. The reaction mixture was boiled in a water bath at 100 °C for 30min and cooled down at 4 °C for 30min. This was followed by addition of 4ml of toluene to the leaf extract, which was then thoroughly mixed. Finally, 1.2ml of the toluene phase was removed for absorbance measurement at 520nm in a UV 2800 spectrophotometer (Unicon). Total soluble sugars in leaves were determined by the modiﬁed phenolsulfuric acid method (Dubois *et al.*, 1951). About 0.1g of leaves were homogenized in 8ml of double-distilled water, and boiled twice in a water bath at 100 °C for 30min. The extract (about 5ml) was transferred to a new microcentrifuge, mixed with 1.5ml of double-distilled water, 1ml of 9% (v/v) phenol, and 5ml of sulfuric acid added, and kept at room temperature for 30min. The absorbance was measured at 485nm in a UV 2800 spectrophotometer (Unicon).

### Flow cytometry of cell cycle progression

T2 generation seeds of *OsRAN1* transgenic rice were sterilized with 0.15% mercuric chloride and germinated on the filter with sterilized water at 28 °C in the dark for 7 d. All of the seedlings of wild-type or *OsRAN1* transgenic rice were assigned in equal quantity and subjected to 28 °C or 4 °C for 12h. Samples of cell nuclei were prepared as described by (Galbraith *et al.*, 1999). Root apical tips (3mm) were excised, immediately chilled on ice, and chopped with a single-edged razor blade in a glass petri dish (diameter, 10cm). Chopping buffer (45mM MgCl_2_, 30 mM sodium citrate, 20mM 4-morpholinepropane sulfonate, and 1mg μl^−1^ Triton X-100, pH 7.0) was used to release the cells from the chopped tissues. The DNA content of individual transgenic cells was determined by flow cytometry. Cell nuclei were stained with 2 μg μl^–1^ 2-(4-amidinophenyl)-6-indolecarbamidine dihydrochloride (DAPI) (Ma *et al.*, 2009). Each sample was prepared three times and subjected to BECKMAN COULTER Moflo-XDP three times. A total of 10 000 nuclei were designed to be measured per analysis.

### Nuclear envelope observation

The transgenic and wild-type seeds were germinated for 7 d and then treated for 0 and 3h at 4 °C. The root tips (2–3mm) were fixed for 5–6h in the fixation buffer (3% glutaraldehyde in 0.1 m PBS, pH 7.2). The materials were washed three or four times with 0.1 m PBS, and fixed in 1% osmic acid under 4 °C overnight. The materials were washed three or four times with 0.1 m PBS, dehydrated with an ethanol series of 30, 50, and 70% (4 °C, overnight), and then 80, 90, 95, 100–100% (30min for every concentration). Ethanol was replaced with acetone (1:1) and infiltrated with acetone and a mixture of resin:acetone:resin=2:1; 1:1; 1:2, for 3h and 100% resin for 12h. The materials were embedded, and polymerized under 60 °C for 24h. Root tips were cut into 50–70nm using an ultramicrotome, the nuclear envelope (NE) was observed by transmission electron microscopy (HITACHI H-7650, Japan).

### Analyses of auxin effects

Col-0 *Arabidopsis* seeds were surface sterilized in 75% ethanol for 1min, followed by 50% (v/v) NaClO solution for 8min, and rinsed in sterile water. Seeds were then placed on plates and vernalized for 72h to synchronize germination. After vernalization, all plates were placed in the same growth chamber and allowed to grow for 10 d to determine the effect of various concentrations of auxin (IAA) on root length and lateral root production (Kim *et al.*, 2001)

## Results

### Identiﬁcation and characterization of the *OsRAN1* gene

**Fig. 1.**
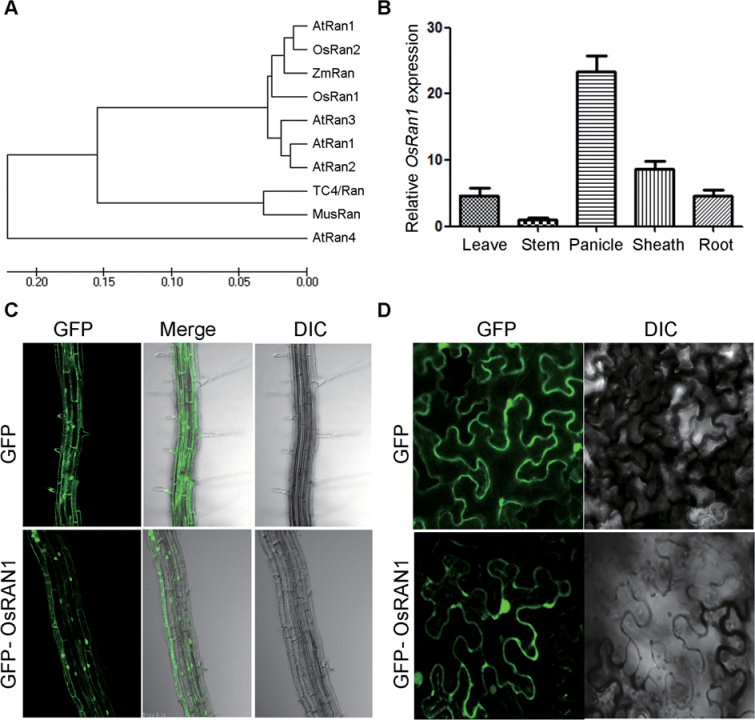
Phylogenetic analysis of plant Ran GTPase, tissue specific expression of *OsRAN1*, and its subcellular localization. (A) Phylogenetic tree of plant Ran GTPases. The tree was constructed with the MEGA5.1 software with amino acid sequences of rice *OsRAN1, OsRAN1*, and other Ran GTPases isolated from wheat (TaRAN1), *Arabidopsis* (AtRAN1, AtRAN2, AtRAN3), human (RAN/TC4), and mouse (MusRAN). (B) Quantitative real-time PCR quantification of *OsRAN1* expression across different rice plant organs. Data are means±SD (*n*=3–5). Panicles, leaves, stems, and roots were harvested from individual 8–9-week-old wild-type plants. Stem expression was set to 1. (C) Subcellular localization of *OsRAN1* in transgenic *Arabidopsis* root cells and tobacco epidermal cells; (d).Transient expression of *OsRAN1:GFP* in tobacco epidermal cells; DIC, differential interference contrast, referring to brightfield images of the cells.

The *OsRAN1* gene was cloned using primers designed according to Accession no. AB015971. *OsRAN1* cDNA was isolated as a full-length coding region and encoding the predicted protein of 221 amino acids. A nucleotide BLAST search (http://blast.ncbi.nlm.nih.gov) was performed against the *Oryza sativa* genome matching the isolated gene to the *Os01g0611100* gene. The predicted protein sequence of OsRAN1 was aligned with related sequences from *Arabidopsis* (AtRan1, AtRan2, AtRan3, and AtRan4), wheat (OsRAN1), human (Ran/TC4), mouse (MusRan), *Zea mays* (ZmRan), and rice (OsRAN2, OsRAN3) (Fig. 1A). The alignment showed 87% sequence homology between OsRAN1 and its human counterpart, and 95% homology between OsRAN1 and OsRAN2 at the amino acid level. The characteristic domains of the Ran proteins, which are known to be involved in GTP-binding and hydrolysis, in addition to the acidic C-terminal domain and the effector-binding domain, are highly conserved in most Ran proteins of various organisms (Ma *et al.*, 2009).

The expression pattern of *OsRAN1* in leaves, root, stem, sheath, and panicle was investigated using quantitative real-time PCR. The expression level was higher in panicle than in other organs examined (Fig.1B). The subcellular localization of *OsRAN1:GFP* was traced to root cells of transgenic *Arabidopsis* steadily overexpressing *OsRAN1* and tobacco epidermal cells expressing transiently. The green fluorescent signal of *OsRAN1:GFP* was detected mainly within the nucleus, with some signals in the hypocotyl cytoplasm (Fig. 1C), whereas the green fluorescent signal was randomly distributed in the cell under the GFP vector control. These findings were consistent with mammalian counterparts, which indicates that Ran is GDP-bound in the cytoplasm during interphase, and GTP-bound in the nucleus (Quimby and Dasso, 2003). In addition, this localization pattern was also observed through transient expression of *OsRAN1:GFP* in tobacco epidermal cells with identical results (Fig. 1D).

### Expression pattern of *OsRAN1* in response to cold, salt, polyethylene glycol, and indoleacetic acid treatment

**Fig. 2.**
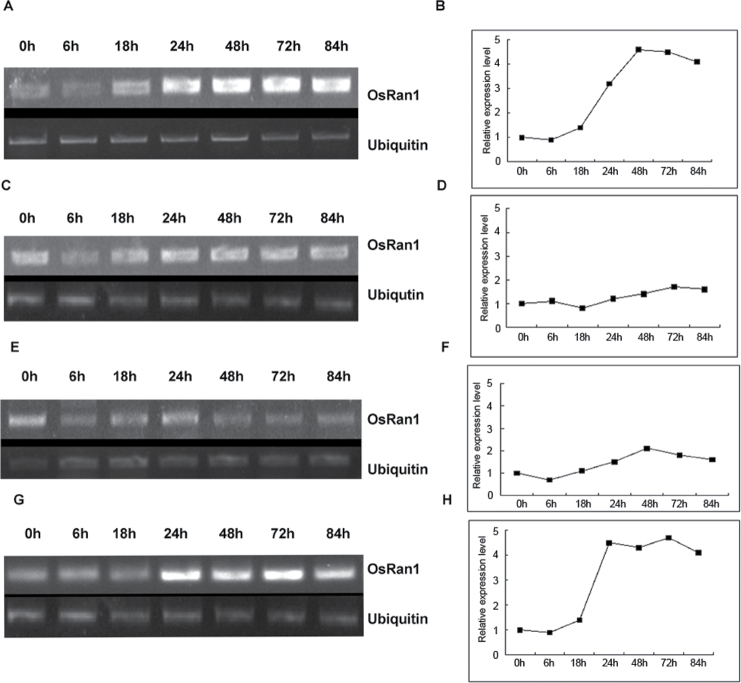
Semi-quantitative RT-PCR analysis of *OsRAN1* expression in stress response. (A, B). Time course of *OsRAN1* expression during cold treatment (4 °C). (C, D) Time course of *OsRAN1* expression during treatment with 150mm NaCl. (E, F) Time course of *OsRAN1* expression during treatment with 10% PEG 6000, a mimic for drought stress. (G, H). Time course of *OsRAN1* expression during treatment with 1 μM IAA. The rice seedlings were germinated and grew for 10 d before they were treated with cold, salt, drought, and IAA stresses. *OsUbiquitin* was used as an internal control.

We investigated the effects of abiotic stress on *OsRAN1* expression using semi-quantitative RT-PCR to monitor the expression pattern of *OsRAN1* in response to different stresses. The transcript level of *OsRAN1* began to increase after 18h of cold treatment and further accumulated up to the peak level at 48h (Fig. 2A, B). Saline stress was induced with NaCl (150mM) (Verslues *et al.*, 2006) increasing the expression of *OsRAN1* after 6h and only slightly thereafter (Fig. 2C, D). We then investigated the effect of osmotic stress on the expression of *OsRAN1*, using 10% polyethylene glycol (Mn 6000; PEG 6000) to mimic osmotic stress. The expression of *OsRAN1* was nearly 2-fold higher than that in the control 48h after treatment, when it decreased again to normal level (Fig. 2E, F). Approximately 1 μM indoleacetic acid (IAA) was used for 84h, with *OsRAN1* levels starting to increase after 24h of treatment and peaking at 72h, with a 5-fold increase (Fig. 2G, H). In conclusion, the data suggest that OsRAN1 predominantly responds to low temperature and IAA treatment compared with salt and drought stress.

### Overexpression of *OsRAN1* increased tiller number and later flowering, and reduced apical dominance and abnormal roots in transgenic *Arabidopsis*

**Fig. 3.**
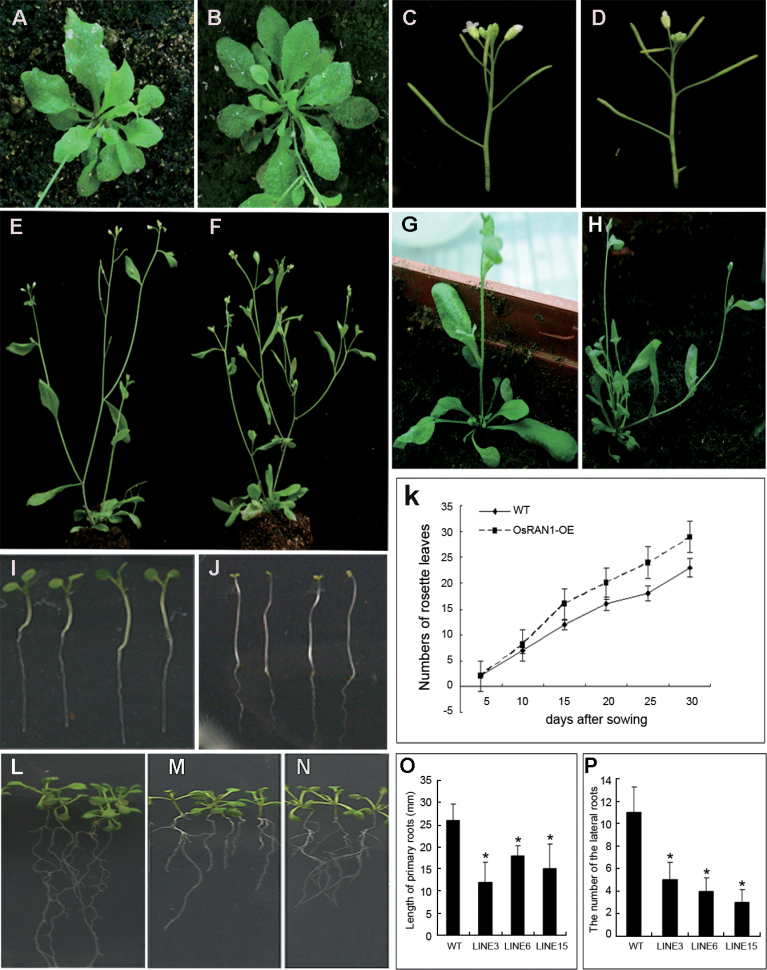
T2 generation phenotypes of different lines in overexpressed *OsRAN1* transgenic *Arabidopsis*. (A) Rosette leaves of wild-type *Arabidopsis* grown for 3 weeks. (B) Increased rosette leaves of transgenic *Arabidopsis* grown for 3 weeks. (C) Apical inflorescence of wild-type *Arabidopsis*. (D) Apical inflorescence including partial abortion of transgenic *Arabidopsis*. (E) Normal floral apical dominance of wild-type *Arabidopsis*. (F) Increased tillery number in 35S-sense *OsRAN1* transgenic mature *Arabidopsis*. (G) Normal phenotype of wild-type *Arabidopsis*. (H) Two branches with rosette leaves in a transgenic plant. (I) Wild-type and transgenic 6-d-old seedlings with elongated hypocotyl in the light. (J) Wild-type and transgenic 6-d-old seeding’s in the dark. (K) Time curve of development of rosette leaves in wide-type and transgenic *Arabidopsis* plants. (L) Normal 10-d-old seeding. (M, N) Transgenic *Arabidopsis* with much fewer lateral roots and shorter main roots. (O) Statistical analysis of wild-type and transgenic lines main root length. (P) Statistical analysis of lateral root number in wild-type and transgenic lines. Asterisk indicates significant difference *P*<0.01.

**Fig. 4.**
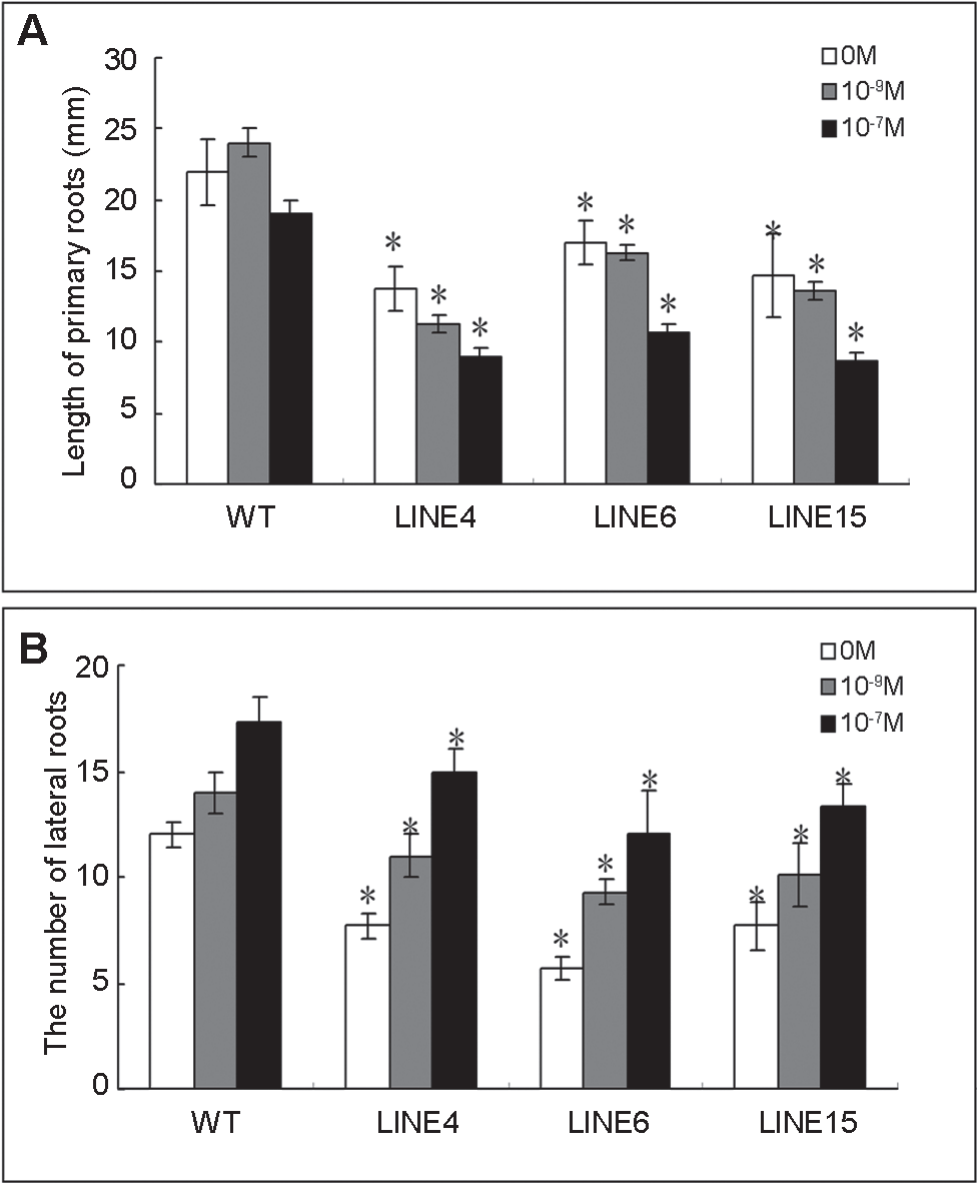
Effect of auxin on root development in transgenic *Arabidopsis* plants. (A) Effect of IAA (10^−9^ or 10^−7^ M) on growth of primary roots in transgenic plants of *Arabidopsis*. The transgenic plants treated with IAA affected primary root growth compared with wild-type plants. (B) Effect of IAA (10^−9^ or 10^−7^ M) on lateral root in transgenic *Arabidopsis* plant. Results are presented as average values±SE from three experiments. More than ten roots were used in each experiment. Asterisk indicates significant difference *P*<0.01.

**Fig. 6.**
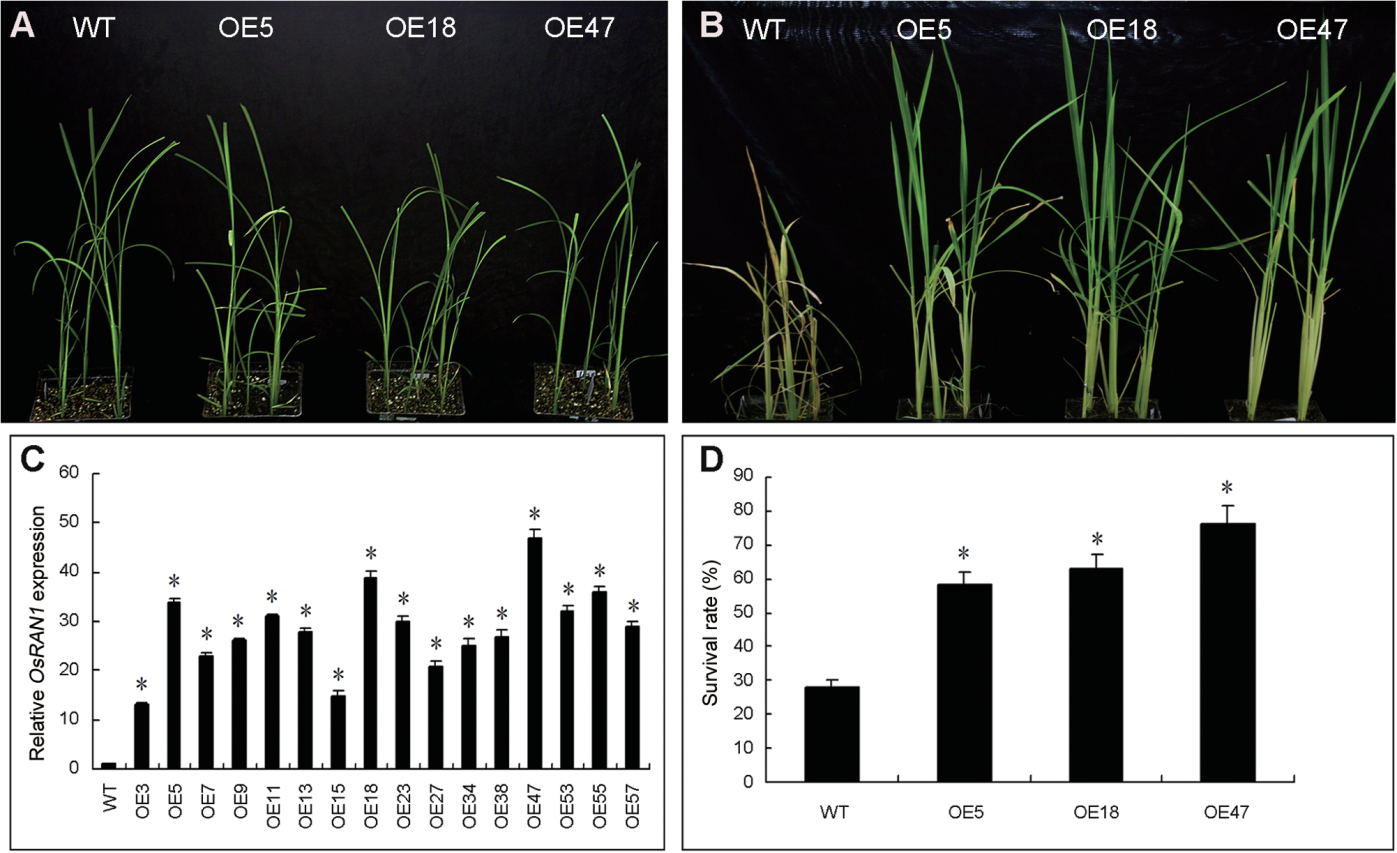
Cold tolerance analysis of transgenic rice overexpressing *OsRAN1*. (A) Two-week-old OE transgenic and WT plants (top photographs) were cold stressed at 4 °C for 84h and then transferred back to the normal condition for recovery. (B) Photographs of representative seedlings of WT and three transgenic lines were taken after 14 d of recovery. (C) Real-time RT-PCR analysis of the expression of *OsRAN1* in transgenic rice relative to that of actin. Data represent means and SE of three replicates. OE3, OE5, OE7, OE9, OE11, OE13, OE15, OE18, OE23, OE27, OE34, OE38, OE47, OE53, OE55, OE57 represent transgenic lines overexpressing (OE) *OsRAN1*. (D) Survival analysis of WT and *OsRAN1* transgenic plants 14 d after cold treatment. Error bars indicate standard deviation and results are from three independent replications of the same experiment. The phenotype was confirmed by further experiments that were repeated more than three times. Asterisk indicates significant difference *P*<0.01. (This figure is available in colour at *JXB* online.)

To analyse the roles of *OsRAN1* in plants further, *OsRAN1* was overexpressed in rice and *Arabidopsis* under the control of the double constitutive cauliflower mosaic virus (CaMV) 35S promoter. Stable inherited homozygous transgenic lines were obtained at the T_2_ generation. In addition, semi-quantitative reverse transcription (RT-PCR) and real-time quantitative PCR (qPCR) were conducted to examine exogenous *OsRAN1* expression in the transgenic *Arabidopsis* and rice lines. As expected, it was found that *OsRAN1* was overexpressed in both transgenic *Arabidopsis* and rice plants (Fig. 6C and Supplementary Fig. S1 available at *JXB* online). Furthermore, we observed the development of the phenotypes. We obtained 25 highly overexpressed lines from 32 independent transgenic lines (Supplementary Fig. S1 available at *JXB* online). Compared with the wild type, all the highly overexpressed transgenic *Arabidopsis* showed distinct phenotypes, such as excess rosette leaves, increased tiller number, longer hypocotyl in the white light, weak apical dominance, and abnormal root development (Fig. 3A–J). The ﬂowers emerged about 4 d later in *OsRAN1* transgenic plants on long days (Fig. 3K). *Arabidopsis* plants overexpressing *OsRAN1* were shorter, with more lateral ﬂoral branches, and partly abortive flowers compared with wild-type plants (Fig. 3C–H). Overall, the apical dominance of transgenic *Arabidopsis* was reduced. Other distinct phenotypes among *OsRAN1* transgenic *Arabidopsis* seedlings related to root development (Fig. 3L–P). Many lines of transgenic seedlings showed a similar phenotype, such as greatly reduced number of lateral roots and stunted primary roots. The number of lateral roots was only 4.4 per plant on average in the transgenic seedlings. In contrast, the wild type showed 10.1 per plant under the same condition. Exogenous applications of IAA addressed the phenotype of fewer lateral roots (Fig. 4). These results demonstrated that *OsRAN1* controls development of shoots and the roots probably by affecting IAA signalling in the transgenic *Arabidopsis*.

### Overexpression of *OsRAN1* increased cell division in rice roots

**Fig. 5.**
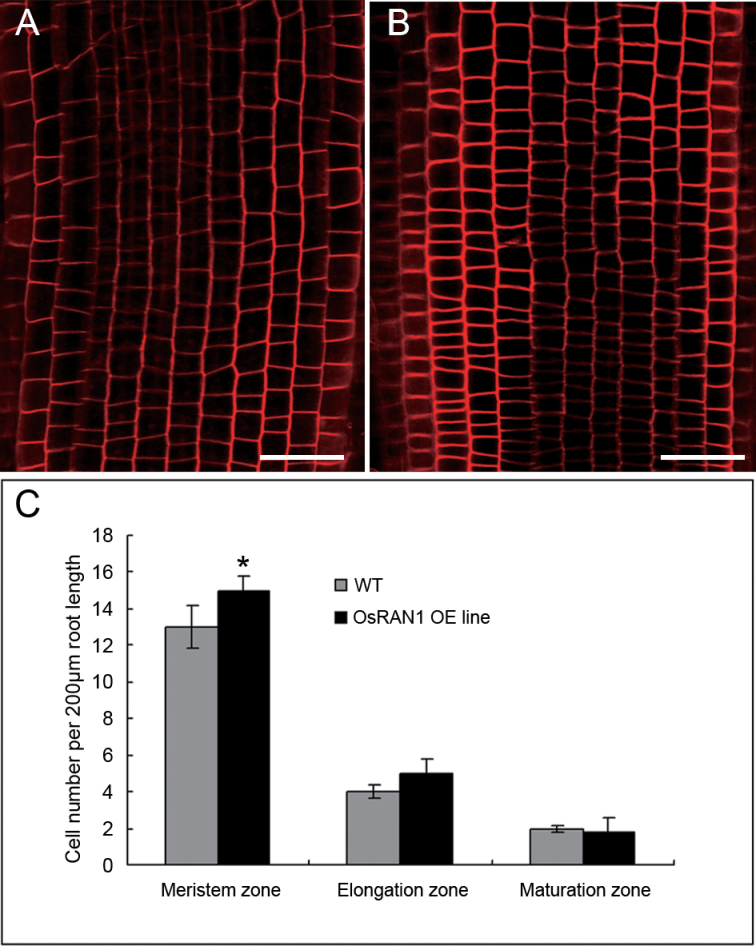
Comparison of cell numbers between transgenic plants and wild-type rice. Primary root meristem zone of primary roots of wild-type (A) and transgenic (B) rice stained with propidium iodide. The number of transgenic rice cells in the meristem was increased over those in the wild type. Bar=50 μm. (C) Cell number in primary roots meristem elongation. Results are presented as mean±SE from three experiments (*n*=5–10). Bar=50 μm. Asterisk indicates significant difference *P*<0.01. (This figure is available in colour at *JXB* online.)

**Fig. 8.**
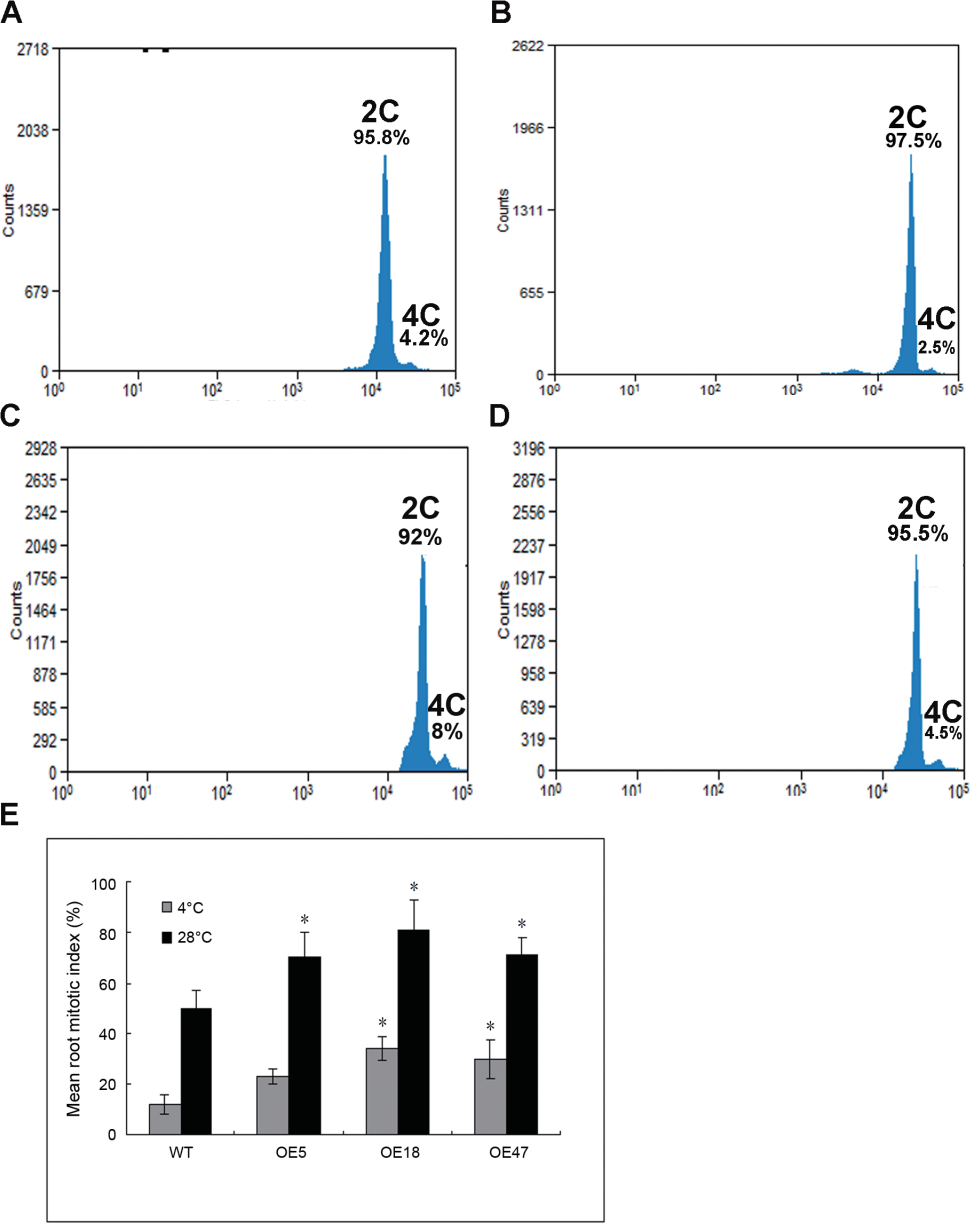
Cell cycle progression and mean root tip mitotic index of wild-type and transgenic rice overexpressing *OsRAN1* during cold stress. (A) The wild-type at 28 °C. (B) The wild-type at 4 °C. (C) Overexpressing line 47 (OE47) of *OsRAN1* transgenic rice at 28 °C. (D) Overexpressing line 47 at 4 °C. Seedlings 7 d after germination were treated with low temperature (4 °C) or room temperature (control, 28 °C) for 12h. Cell nuclei (10 000) taken from the root apical meristem were stained with DAPI and analysed by flow cytometry. 2C and 4C represent the DAPI signals that correspond to nuclei with different DNA contents. (E) Cell mitotic index in root apical meristem (RAM) in rice. The error bars show SE and are from three independent replications of the same experiment. Six root tips were analysed in every replicate. Asterisk indicates significant difference *P*<0.01. (This figure is available in colour at *JXB* online.)

We further examined the phenotypes in the OsRAN1 overexpressed lines of rice. The transgenic rice plants showed a tiller number up to 9.3 per plant on average. In contrast, wild-type rice had fewer tillers, about 7.3 per plant (Table 1). The *OsRAN1* overexpressed rice plants were shorter with more tillering branches. These results suggest that *OsRAN1* expression affected tiller initiation in the shoot meristem. Furthermore, the roots of transgenic rice contain many small and tightly arrayed cells emerging from the meristem (Fig. 5). This information, combined with the results of the increased 4C DNA in the *OsRAN1* overexpression line (Fig. 8A–D), indicates that *OsRAN1* is involved in meristem cell proliferation in the root. *OsRAN1* probably regulates cell division, particularly in the root tip meristem.

**Table 1.** Tiller number in 35S-sense *OsRAN1* transgenic mature rice

Rice plant line	Tillery No.	Plant height (cm)	Panicle No.
Wild type	7.25±0.83	91.61±2.43	6.20±2.22
OE 5	9.2±1.31^a^	85.8±3.17^a^	7.20±0.84
OE 18	9.4±1.44^a^	84.3±5.18^a^	7.40±2.08
OE 47	9.4±4.19^a^	83.9±2.32^a^	8.10±1.53^a^

^a^ Significant differences, *P*<0.01. (SE of the mean is based on eight mature plants)

### Overexpression of *OsRAN1* increased cold tolerance in transgenic rice and *Arabidopsis*

To test the possible role of *OsRAN1* overexpression in cold tolerance of rice, the seedlings of T2 transgenic lines and wild type at trefoil stage were exposed to cold stress at 4 °C for 84h. The plants were then removed to the greenhouse to recover at 28 °C. After 14 d recovery under normal conditions, the survival rates of three transgenic lines were between 71% and 78%, whereas the survival rate for the wild type was only 26 % (Fig. 6). We further examined the freezing tolerance of *Arabidopsis* overexpressing *OsRAN1*. The results showed that transgenic lines 3, 5, and 15 have higher freezing tolerance after 3 d cold acclimation. The survival rate of these three lines after 2 weeks of recovery was 52%, 68%, and 60%, whereas the survival rate of the wild type was 32% (Supplementary Fig. S2 available at *JXB* online). Therefore, we concluded that *OsRAN1* overexpression increases the freezing tolerance of transgenic rice and *Arabidopsis*.

### Increased Pro and soluble sugar contents in *OsRAN1* transgenic plants under cold stress

**Fig. 7.**
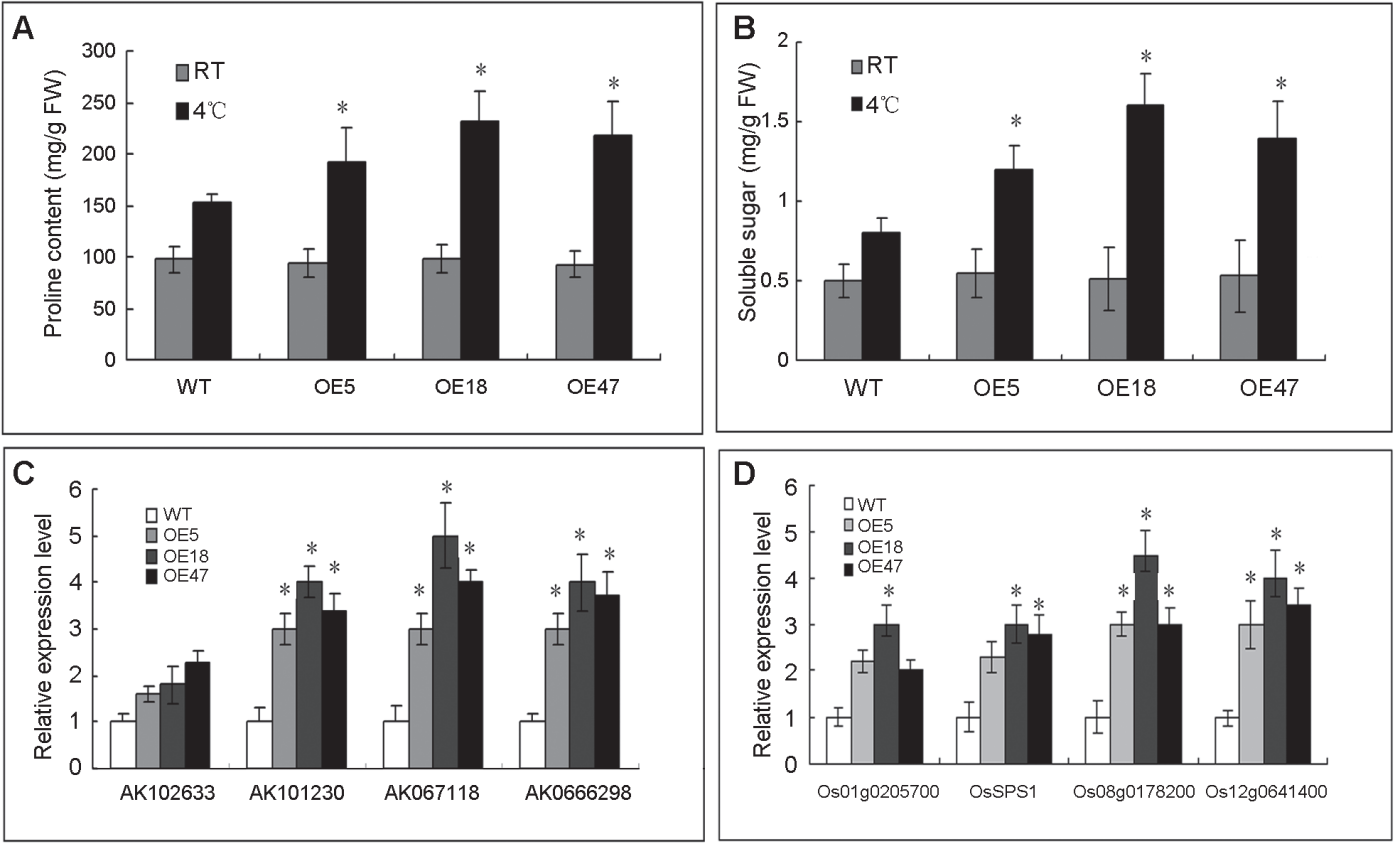
Contents of soluble sugars and free Pro in transgenic rice plants overexpressing *OsRAN1* compared with the wild-type plants under normal and cold stress. Free Pro (A) and soluble sugar (B) contents in the *OsRAN1*-overexpressing plants under cold stress (4 °C) for 3 d. Values are means±SD (*n*=4). FW, fresh weight. (C) Expression levels of putative Pro synthesis genes (*AK102633* and *AK101230*) and Pro transporter genes (*AK067118* and *AK0666298*) in cold-stressed (4 °C for 1 d) *OsRAN1* transgenic plants by real-time PCR. (D) Expression levels of putative sugar synthesis relative genes (Os01g0205700 and OsSPS1) and sugar putative transporter genes (Os08g0178200 and Os12g0641400) and two putative sugar transporter genes (Os08g0178200 and Os12g0641400) in cold-stressed (4 °C for 1 d) *OsRAN1* transgenic plants by real-time PCR. The bars represent three repeats. Asterisk indicates significant difference *P*<0.01.

Plant adaptation to environmental stresses is often associated with metabolic adjustment, such as accumulation of Pro and soluble sugars (Abraham *et al.*, 2003). To investigate the physiological basis for the improved stress tolerance in transgenic rice, we measured the Pro and soluble sugar content in plants that overexpressed the *OsRAN1* gene and the wild type under normal growth and stress conditions. Under normal growth conditions (28 °C), the levels of cellular free Pro did not differ between wild-type and transgenic rice, at quantities ranging between 92 and 98mg fresh weight (Fig. 7A). In contrast, after cold treatment (4 °C), the levels of free Pro in *OsRAN1* transgenic rice increased substantially, with more than 178mg g^–1^ (207mg g^–1^,198mg g^–1^) fresh weight compared with 146mg g^–1^ fresh weight in the wild-type plants. The levels of free sugar in *OsRAN1* transgenic rice increased to 1.1mg g^–1^ (1. 6mg g^–1^,1.4mg g^–1^) fresh weight compared with 0.85mg g^–1^ fresh weight in the wild-type plants after cold treatment, although the levels of cell-free sugar did not differ between the two types of rice (Fig.7B).

We further measured the expression levels of two putative carboxylate synthetase genes (*AK102633* and *AK101230*) and two putative Pro transporter genes (*AK067118* and *AK0666298*) in the *OsRAN1* transgenic plants subjected to cold stress. As show in Figure 7C, *AK067118*, *AK0666298*, and *AK101230* had higher expression level (3- to 5-fold) in the *OsRAN1* overexpressed lines than in the wild type after cold treatment. We also measured the expression level of putative sugar synthetase relative genes Os01g0205700 and OsSPS1 and two putative sugar transporter genes (Os08g0178200 and Os12g0641400) in the *OsRAN1* transgenic plants under cold stress. Genes in the *OsRAN1* overexpressed lines had higher expression level (2-fold–4.5-fold) than the wild type (Fig.7D).

### Cell cycle progression in transgenic rice lines

We monitored the mitotic index of AtRAN1-overexpressed lines under cold conditions. Mitotic index is defined as the ratio between the number of cells in mitosis and the total number of cells, which is used as a measure for the proliferation status of a cell population. Flow cytometry showed that the 4C DNA content of *OsRAN1*-overexpressed lines increased in comparison with the wild type under normal (28 °C) and cold (4 °C) conditions (Fig. 8A–D). Under the normal conditions, the index for the transgenic lines was 61–78% compared with that of the wild-type 46%. Even at 4 °C, the index for the transgenic lines was 21–31% compared with that of the wild-type 14% (Fig. 8E), although both the transgenic lines and the wild type showed a significantly decreased index at this temperature. Thus, the overexpressing lines possessed more cells in the proliferation, especially under the cold condition. Therefore, we conclude that *OsRAN1* overexpression increased mitosis.

### Overexpression of *OsRAN1* promoted the formation of intact nuclear envelope under cold stress

**Fig. 9.**
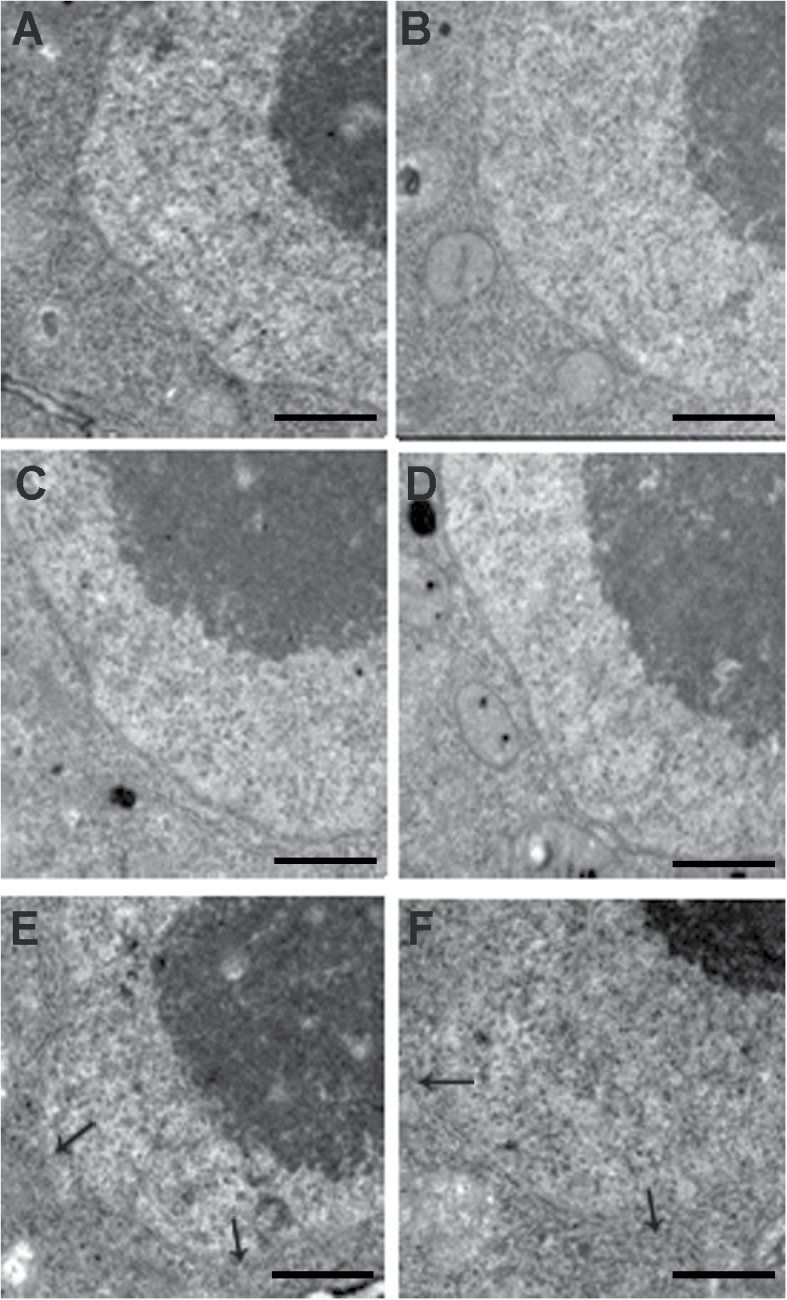
Morphologies changes of the nuclear envelope in the wild type (WT) and transgenic line under cold stress. Nuclear envelope of the WT (A) and *OsRAN1*-overexpressing line 47 (B) under normal conditions (28 °C); nuclear envelope of the WT (E, F) and *OsRAN1*-overexpressing line (C, D) after 3h treatment at 4 °C. Six root tips were observed in every condition. The root tips were transversely cut in the meristematic zones. Arrows indicate the nuclear envelope. Bars=500nm.

Next, we observed the nuclear envelope (NE) of root tip cells of the overexpressed transgenic line 47 and wild-type rice under normal and cold conditions. Under the normal condition (28 °C), NEs of the transgenic line and wild type were all intact with no obvious difference in morphology (Fig. 9A, B). After treatment at 4 °C for 4h, about 70% cells in wild type showed obscured or partly dissociated double membranes (Fig. 9E, F). However, most cells in the overexpressed lines formed an intact NE under cold stress (Fig. 9C, D), with cells showing partially dissociated NE. The results suggested that overexpression of *OsRAN1* may promote the formation of an intact NE under cold stress.

## Discussion

### *OsRAN1* in cell cycle regulation

Several studies indicate that the small GTPase called Ras-related nuclear protein (Ran) is a central regulator of several cell cycle events. In the 1990s, scientists first discovered that Ran was an essential factor for protein import into the nucleus during interphase. In subsequent studies, they discovered that Ran played a role in spindle assembly and nuclear envelope formation during mitosis. Ran seems to modulate these vastly different cellular processes through a gradient mechanism, wherein active Ran is more concentrated around its primary site of action and less concentrated elsewhere (Clarke and Zhang, 2001). Overexpression of various wild-type Ran homologues from plants, including tomato and tobacco Ran, suppressed the *pim1* mutant phenotypes in yeast (Lee *et al.*, 1993; Merkle *et al.*, 1994), which suggests that the role of Ran GTPase may be conserved in cell cycle regulation. The precise details associated with specific processes during cell cycle progression have yet to be delineated (Rossi and Varotto, 2002). Plant meristematic cells do not undergo cellular expansion and are relatively uniform in size. The size of the meristematic cells of the primary root tip was reduced on average in our *OsRAN1*-overexpressed plants. The root-tip meristem of *OsRAN1*-overexpressed plants contained smaller cells than those in the wild type, thus increasing the total number of meristematic cells. In brief, our study reveals that *OsRAN1* was involved in cell proliferation in the root. *OsRAN1* overexpression enhanced the root tip mitotic index of transgenic rice (Fig. 8E), further increasing the cold tolerance. Previous research demonstrated that OsRAN1 and TaRAN1, sharing high identity at the amino acid level, were also increased in cold tolerance in transgenic rice plants (Chen *et al.*, 2011). Another study conducted by our laboratory in *Arabidopsis* showed that *atran1 atran3* double mutants have increased freezing sensitivity, whereas the *atran1* or *atran3* signal mutant in *Arabidopsis* did not have increased freezing sensitivity (data not shown). These results indicate that Ran GTPase regulates cold stress redundantly. Thus, Ran homologues may have well-conserved functionality in plants, yeast, and animals during evolution.

### Functional analysis of *OsRAN1* suggests a role in auxin signal transduction; exogenous application of IAA partly repairs the transgenic root defects

Response to IAA in the lateral root initiation involves multiple root and shoot phenotypes commonly associated with auxin mutants (Berleth *et al.*, 2000). We tested whether *OsRAN1* transgenic *Arabidopsis* root development defects were affected by auxin by supplementing the growth medium with exogenous IAA The results indicate that IAA typically promotes the lateral root initiation but have no obvious effects on primary root length in transgenic *Arabidopsis* (Fig. 4). In the auxin-signalling pathways, suppressors of auxin action block the expression of auxin-induced genes in the nucleus (Ulmasov *et al.*, 1999). Ran proteins play an important role in nuclear transport. Overexpression of OsRAN1 protein might result in an abnormal or reduced rate of transport of important protein modulators to the nucleus. The *OsRAN1*-overexpressed transgenic plants are partly recovered by auxin, supporting the hypothesis that Ran was regulated by auxin and was involved in the auxin-mediated signalling pathway.

### *OsRAN1* enhanced cold tolerance by maintaining cell division and regulating the intact NE under cold stress in rice

Studies have shown that cell division is closely related to stress tolerance in plants. *Arabidopsis* plants constitutively overexpressing *HAL3a* (Halotolerance gene 3a) showed improved growth, as well as salt and osmotic tolerance in *Arabidopsis* and rice (Espinosa-Ruiz *et al.*, 1999; Rubio *et al.*, 2006). Transgenic rice lines overexpressing *OsMYB3R-2* (R1R2R3 MYB gene) and *OsCycB1;1* (Cyclin B gene 1;1) exhibited enhanced cold tolerance, which indicates that ongoing mitosis enhances cold resistance in plants (Ma *et al.*, 2009). Our results indicate that the expression of *OsRAN1 was* up-regulated under cold stress (Fig. 2). Also, cellular Pro and free sugar level are involved in *OsRAN1* transgenic rice cold tolerance. These results were similar to the alterations observed in other transgenic rice overexpressing cold resistance genes, such as *OsNAC6*, *OsCIPK03*, and *OsCIPK12* (Nakashima *et al.*, 2006; Xiang *et al.*, 2007). *OsRAN1* overexpression could maintain a high mean root mitotic index under cold stress and cell cycle progression (Fig. 8), suggesting that *OsRAN1* functions as a regulator in the cold signalling pathway in rice.

Ran plays a pivotal role in the regulation of the nuclear envelope assembly under the cooperation of its binding proteins RanGAP1, RCC1, importins, etc. LBR (lamin B receptor), a chromatin and lamin B binding protein in the inner nuclear membrane, targets the membrane precursor vesicles to the chromatin mediated by importin β during NE assembly. Ran GTPase also promotes nuclear pore organization at the end of mitosis in *Xenopus* egg extracts (Zhang *et al.*, 2002). Despite the previous report of the function of plant Ran GTPase *OsRAN2* in NE assembly, our NE observations indicated that *OsRAN1* overexpression also promoted the formation of an intact NE under cold stress (Fig. 9). By combining our results with those of previous studies, we speculate that both *OsRAN1* and *OsRAN2* may be involved in the organization of normal NE structures at the end of mitosis during the cold condition. In conclusion, *OsRAN1* overexpression could enhance cold tolerance by maintaining cell division ability and NE assembly under cold stress in rice.

## Supplementary data

Supplementary data are available at *JXB* online

Table S1. Primers used in plasmid constructions transgenes Primers.

Table S2. Primers used in RT-PCR in detecting gene expression level in transgenic plants.

Table S3. Gene-specific primers used in qPCR experiments.

Figure S1. Transgenic *OsRAN1 Arabidopsis* expression pattern.

Figure S2. Transgenic *OsRAN1 Arabidopsis* increased freezing tolerance after cold acclimation.

Supplementary Data
