# Supplementary material for: RAN1 is involved in plant cold resistance and development in rice (Oryza sativa)
Source: J Exp Bot. 2014 Apr 30;65(12):3277–87. doi: 10.1093/jxb/eru178 (PMC4071843; doi:10.1093/jxb/eru178)
Supplement: Supplementary Data [file supp_65_12_3277__index.html]

 RAN1 is involved in plant cold resistance and development in rice (Oryza sativa) — RAN1 is involved in plant cold resistance and development in rice (Oryza sativa) — Supplementary Data 

# *RAN1* is involved in plant cold resistance and development in rice (*Oryza sativa*)

## Supplementary Data

Data files

**Files in this Data Supplement:**

- Supplementary Data - Supplementary Data
