# Supplementary material for: RAN1 is involved in plant cold resistance and development in rice (Oryza sativa)
Source: J Exp Bot. 2014 Apr 30;65(12):3277–87. doi: 10.1093/jxb/eru178 (PMC4071843; doi:10.1093/jxb/eru178)
Supplement: Supplementary Data [file supp_eru178_jexbot120527_file001.pdf]

***OsRAN1* is involved in plant cold resistance and development**

Peipei Xu and Weiming Cai

Supplementary Data

Table S1. Primers used in plasmid constructions Transgenes Primers

Transgenes	Primers (Sequence 5’-3’)
double 35S:OsRAN1	5’- CCAAGCTTATGGCGCTCCCGAATC-3’
	5’-CGGATCCCTACTCGATCAGGTCATCGTC-3’
double35S:OsRAN1:GFP	5’- CCAAGCTTATGGCGCTCCCGAATC-3’[a]
	5’-CGGATCCCTCGATCAGGTCATCGTC-3’[a]
	5’-GGTACCATGGTAGATCTGACTA-3’[b]
	5’GAGCTCTCACACGTGGTGGTGGT-3’[b]

Underlined are restriction digestion sites.
a: specific primers for OsRAN1;
b: specific primers for GFP.

Table S2. Primers used in RT-PCR in detecting gene expression level in transgenic plants

Genes	Accession	Primers
OsRAN1	AB015971	5’- ATGGCGCTCCCGAATC-3’ [a]
		5’-CTACTCGATCAGGTCATCG-3’ [a]
OsRAN1	AB015971	5’- CGATGACCTGATCGAGTA-3’ [b]
		5’- GAGAGCGGATCATACACA-3’ [b]
AtACTIN2	NM_112764	5’-TGTGCCAATCTACGAGGGTTT-3’
		5’-ATTTCTTTGCTCATACGGTCAG-3’
OsUbiquitin (UBI)	D12776	5’-GACGGACGCACCCTGGCTGAC-3’
		5’-TGCTGCCAATTACCATATACCA-3’
Hygromycin Phosphotransferase (HPT)	K01193	5’-TGTCCTGCGGGTAAATAGC-3’
		5’-TGCTCCATACAAGCCAACC-3’

a: primers designed according to OsRAN1 ORF used for gene expression level detected in Arabidopsis;
b: primers designed according to 3’-UTR of OsRAN1 used for gene expression level detected in rice.

Table S3. Gene-specific primers used in qPCR experiments.

Genes	Primers (Sequence 5’-3’)
AtACTIN2	5’-TTCCCGTTCTGCGGTAGTGG-3’
	5’-CCGGTATTGTGCTCGATTCTG-3’
OsUbiquitin	5’-GACGGACGCACC CTGGCTGACTAC -3’
	5’-TGC TGCCAATTACCATATACCACGAC-3’
OsRAN1	5’-GCGCTCCCGAATCAGCAGACG-3’
	5’-AGAAGCGGATCTTGCCGCAGTT-3’
AK102633	5’-CTCAAATCAAGGCGTCAACTAAGA-3’
	5’-TTTGTCAATATATA-CGTGGCATATACCA-3’
AK101230	5’-CGCCCCTCCCCGTATCT-3’
	5’-AGGAATGCGGCAACAAGTG-3’
AK067118	5’-AGGGACGATGGAGT-TCTAAAGCT-3’
	5’-GGGATTCCAAAGGCAAAAAGA-3’
AK0666298	5’-GAGGAGGCTACCTGACTGTCAAC-3’
	5’-GCTCATGAAGTCGCC-AAGGA-3’
Os01g0205700	5’-GGTTCCTGGTGAACCGAACA-3’
	5’-AGCTGGTCAACAGCACTCTC-3’
OsSPS1	5’-CAACTGTTAGCATTCACAAGGTG-3’
	5’-CGCCTAATCTTGGAAGATTGAAAAC-3’
Os08g0178200	5’-CTCATCCCGTGCTTCCAACA-3’
	5’-CGGAGATGATCGAGACGACG-3’
Os12g0641400	5’-GTCTACCTCGTCGGCTTCTG-3’
	5’-CACTGTATGCTCCAGTGGCA-3’

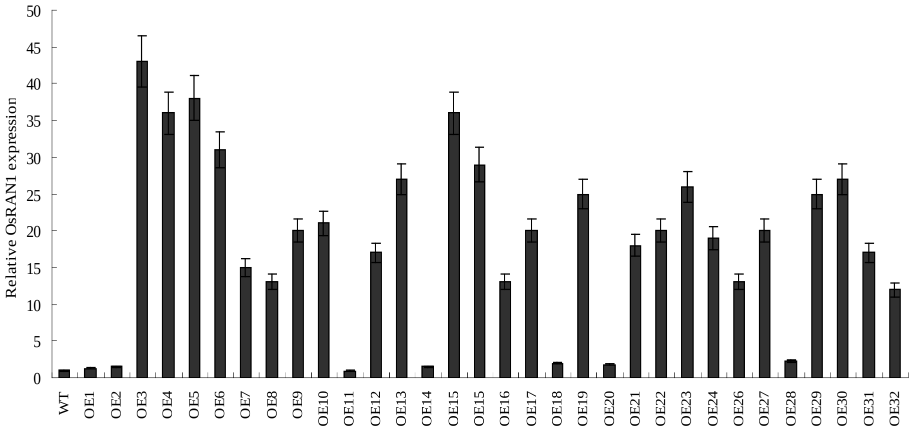

Figure S1. Transgenic OsRAN1 Arabidopsis expression pattern

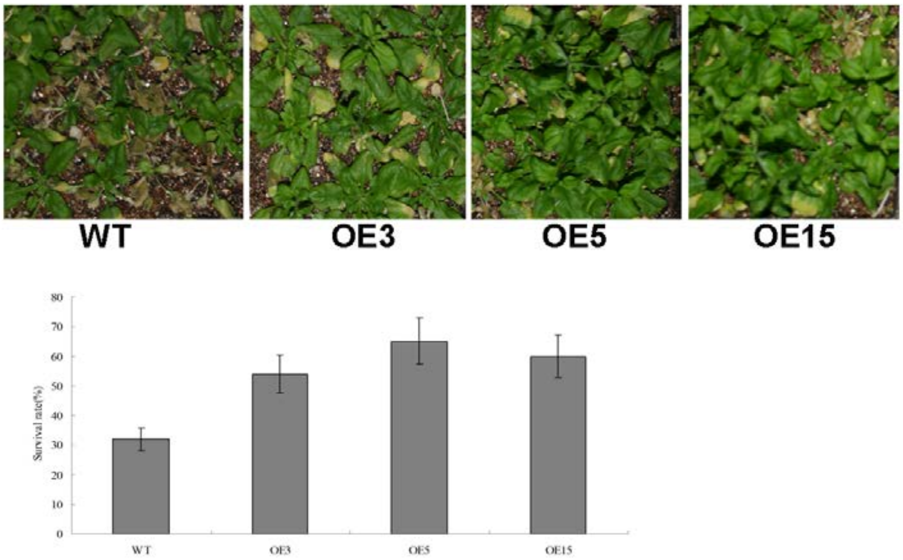

Figure S2. Transgenic OsRAN1 Arabidopsis increased freezing tolerance after cold accumulation

3 weeks old Arabidopsis seedings were cold accumulated at -4°C for 3 days, then transferred to -10°C freezing treatment for 6 hour, photos were taken after 2 weeks recovery at 22°C.
